# High levels of osteoprotegerin are associated with coronary artery calcification in patients suspected of a chronic coronary syndrome

**DOI:** 10.1038/s41598-021-98177-4

**Published:** 2021-09-23

**Authors:** Mirthe Dekker, Farahnaz Waissi, Max J. M. Silvis, Joelle V. Bennekom, Arjan H. Schoneveld, Robbert J. de Winter, Ivana Isgum, Nikolas Lessmann, Birgitta K. Velthuis, Gerard Pasterkamp, Arend Mosterd, Leo Timmers, Dominique P. V. de Kleijn

**Affiliations:** 1 Department of Vascular Surgery (G04129), University Medical Centre Utrecht, Heidelberglaan 100, 3584 CX Utrecht, The Netherlands; 2 Department of Cardiology, Amsterdam University Medical Centres, Amsterdam, The Netherlands; 3 Department of Cardiology, University Medical Centre Utrecht, Utrecht, The Netherlands; 4 Central Diagnostic Laboratory, Arcadia, University Medical Centre Utrecht, Utrecht, The Netherlands; 5 Department of Radiology and Nuclear Medicine, Amsterdam University Medical Centers, Amsterdam, The Netherlands; 6 Department of Radiology and Nuclear Medicine, Radboud University Medical Centre, Nijmegen, The Netherlands; 7 Department of Radiology, University Medical Centre Utrecht, Utrecht, The Netherlands; 8 Department of Clinical Chemistry and Haematology, University Medical Centre Utrecht, Utrecht, The Netherlands; 9 Department of Cardiology, Meander Medical Center Amersfoort, Amersfoort, The Netherlands; 10 Department of Cardiology, St. Antonius Hospital Nieuwegein, Nieuwegein, The Netherlands; 11 Netherlands Heart Institute, Utrecht, The Netherlands

**Keywords:** Cardiovascular biology, Predictive markers, Prognostic markers, Imaging, Isolation, separation and purification, Proteomic analysis

## Abstract

Plasma osteoprotegerin (OPG) and vascular smooth muscle cell (VSMC) derived extracellular vesicles (EVs) are important regulators in the process of vascular calcification (VC). In population studies, high levels of OPG are associated with events. In animal studies, however, high OPG levels result in reduction of VC. VSMC-derived EVs are assumed to be responsible for OPG transport and VC but this role has not been studied. For this, we investigated the association between OPG in plasma and circulating EVs with coronary artery calcium (CAC) as surrogate for VC in symptomatic patients. We retrospectively assessed 742 patients undergoing myocardial perfusion imaging (MPI). CAC scores were determined on the MPI-CT images using a previously developed automated algorithm. Levels of OPG were quantified in plasma and two EV-subpopulations (LDL and TEX), using an electrochemiluminescence immunoassay. Circulating levels of OPG were independently associated with CAC scores in plasma; OR 1.39 (95% CI 1.17–1.65), and both EV populations; EV-LDL; OR 1.51 (95% CI 1.27–1.80) and EV-TEX; OR 1.21 (95% CI 1.02–1.42). High levels of OPG in plasma were independently associated with CAC scores in this symptomatic patient cohort. High levels of EV-derived OPG showed the same positive association with CAC scores, suggesting that EV-derived OPG mirrors the same pathophysiological process as plasma OPG.

## Introduction

Osteoprotegerin (OPG) is a glycoprotein of the tumor necrosis factor receptor family^1,2^. The main function of OPG is to inhibit osteogenesis by preventing the binding of the receptor activator of nuclear factor-κB ligand (RANKL) to its natural receptor activator nuclear factor-κB (RANK)^3^. The RANKL/RANK complex normally results in differentiation of osteoclasts and osteogenesis^3,4^. OPG is therefore important in maintaining the balance between bone formation and resorption^5^.

Additional to its function in bone metabolism, OPG is also implicated in cardiovascular diseases (CVDs)^6^. OPG is thought to be involved in the process of vascular calcification (VC). Experimental studies showed the presence of OPG within the vessel wall in the media and intima, and also in the fibrous cap of atherosclerotic lesions^7^. Animal studies showed more VC in mice lacking OPG than mice without OPG-deficiency^8,9^. Furthermore, administration of OPG to atherosclerotic mice deficient for the LDL receptor led to less calcified plaques compared to the placebo mice^10^. In contrast, human studies show that high levels of plasma OPG are associated with a higher risk of future events^11,12^. Although the exact role remains unclear, it might be that plasma OPG is produced as response to VC to protect against progression rather than preventing it^13,14^.

The discrepancy between the presumed role of OPG in mice studies compared to large population studies could potentially be found in extracellular vesicles (EVs). EVs are bilayer lipid membranes containing bioactive content (nucleic acid, proteins and lipids)^15^. EVs are often referred to as “the liquid biopsy”, and considered as cell–cell communicator^16^. Almost all cell types are able to produce EVs^16^.

EVs derived from vascular smooth muscle cells (VSMCs) are thought to be involved in VC, and contain calcification inhibitors such as OPG and matrix GLA-protein to regulate the micro-environment^17,18^. In pathological circumstances VSMC-derived EVs become microvascular calcified structures that form the start of advanced calcified plaques. In these calcified plaques OPG was found near these VSMC-derived EVs suggesting a role in transportation of OPG by EVs. EVs are often analyzed by total number of EVs, however their content might be also informative^19^. We previously found that EVs can be separated based on size and density, potentially reflecting pathophysiological phenomenon. Despite OPG is associated with EVs, levels of plasma OPG and/or EV-derived OPG and its association with VC has not been studied.

Coronary artery calcium (CAC) score measured with coronary CT is used as surrogate for VC^20^. The CAC score has been shown to be an excellent predictor of major adverse cardiovascular events (MACE), as well as a risk stratifying tool in patients suspected of chronic coronary syndrome (CCS)^21–23^.

The relationship between OPG and CAC is studied in asymptomatic population-based studies as well as patients with renal failure or diabetes mellitus^24–29^. However, little is known about the association between CAC scores and levels of OPG in a symptomatic cohort. Neither do we know if OPG in circulating EVs provides additional information to plasma levels. In this study, we investigate the association between CAC, plasma OPG and circulating EV-derived OPG in two subsets of EVs in patients suspected of CCS.

## Methods

### Study cohort

We will perform a retrospective analysis on the prospectively collected MYOMARKER study cohort. The MYOMARKER (MYOcardial ischemia detection by circulation bioMARKERs) is a prospective single center cohort study of consecutively enrolled patients who underwent myocardial perfusion imaging (MPI) with ^82^Rb-PET/CT because of chest pain suspected for CCS. All patients were aged > 18 years and included between August 2014 and September 2016 in the Meander Medical Center, the Netherlands. The study (NL5078) was approved by the Medical Ethics Committee-United (MEC U), in accordance with the Declaration of Helsinki. Written informed consent was obtained from all patients, more details on the study protocol have been published previously^30^. For the purpose of this study all patients with a history of a percutaneous coronary intervention or coronary artery bypass grafting were excluded.

### Study protocol

**Figure 1:**
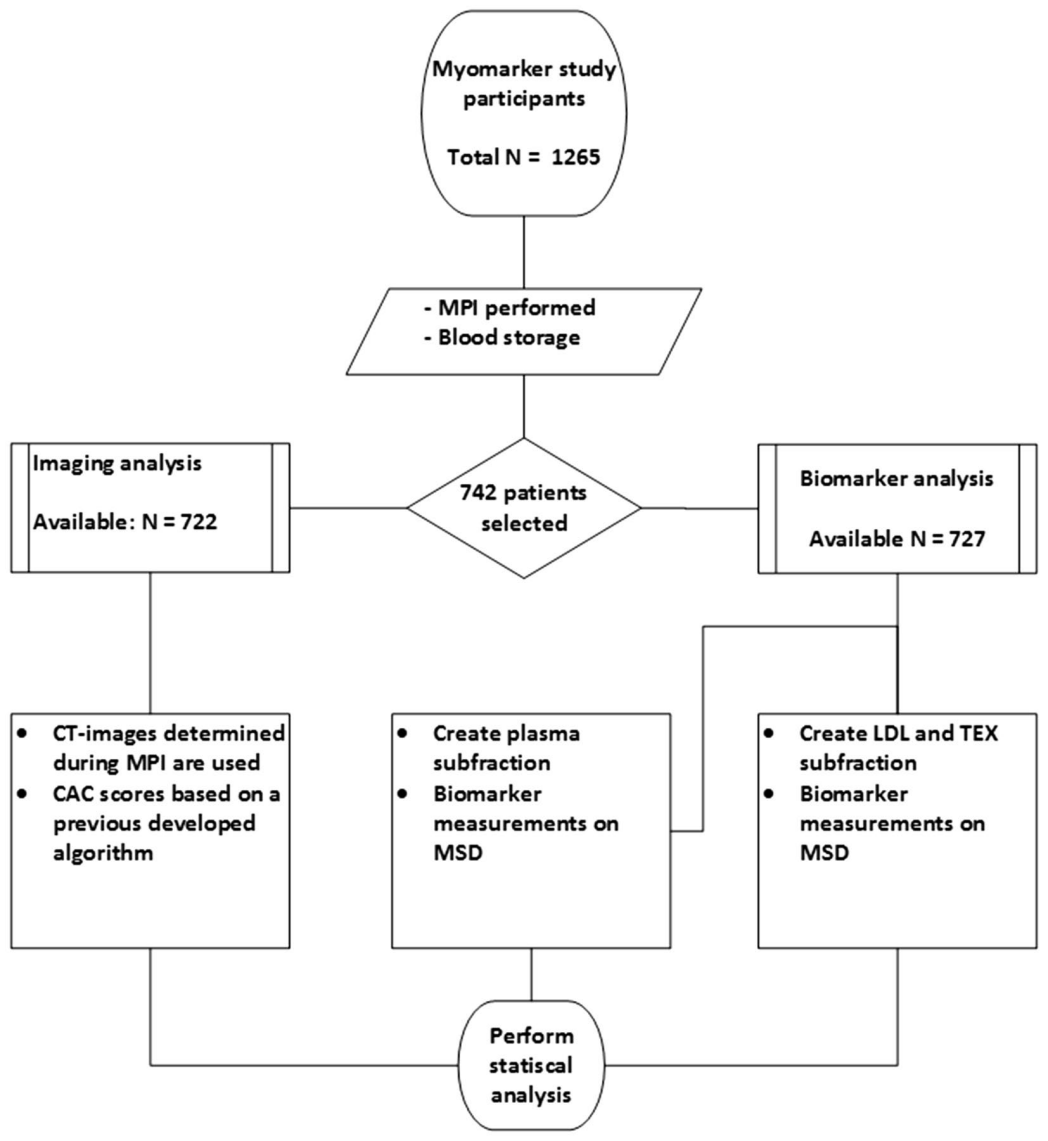
Study protocol. *MPI* myocardial perfusion imaging, *CAC* coronary artery calcification, *MSD* MesoScaleDiscovery platform. LDL and TEX refer to extracellular subfractions.

Levels of OPG were measured in previously collected blood samples. Venous blood was collected in EDTA tubes just before the MPI was performed. The samples were centrifuged 10 min at 1950×*g* at room temperature (RT) within 30 min after they were collected. After centrifugation all samples were aliquoted and directly stored at − 80 °C. Additional to the protein measurements CAC scores were obtained. For an overview of this study protocol see also Fig. 1.

#### Study protocol—extracellular vesicles isolation

Levels of OPG were measured in both plasma as well as in EVs. For this, two EV subpopulations were isolated. The isolation was performed as described in previous publications^30,31^. In brief, a subset of EVs co-precipitate with low-density lipid particles (LDL) which allows separation. Magnetic beads were therefore added for both subpopulations (nanomag^®^-D plain voor LDL and nanomag^®^-D PET-OH for TEX). For the sequential isolation of the EV-LDL subpopulation Dextran Sulphate (DS, 0.05%, MP biomedicals) was used in combination with Manganese II Chloride (MnCl2, 0.05 M, Sigma-Aldrich) (EV-TEX). The TEX subpopulation was precipitated with Xtractt buffer (1:4, Cavadis BV). Subsequently, a bio-plex handheld magnet was used. The remaining pellet containing the EV subpopulations was separated from magnetic bead debris with centrifugation, after removal lysis buffer was added to study the OPG levels carried within the EV subpopulations.

#### Study protocol—extracellular vesicle quantification

Levels of OPG were quantified in plasma and the EV-LDL and EV-TEX subpopulations using an electrochemiluminescence immunoassay (Meso Scale Discovery, MSD) following manufacturers protocol. In short; MSD GOLD Small Spot Streptavidine plates were coated O/N at 4 °C with OPG antibodies (MSD R-Plex human OPG antibody set, F21ZK). After washing three times with 150 µL wash buffer (0.2% Tween-20 in PBS) per well, the coated plates were blocked with blocking buffer A (MSD) for 1 h at RT. Subsequently, plates were then washed as described before and 50 µL diluted plasma or protein lysate (twofold) from the EV subfractions, blancs or calibrators were added to designated wells and incubated for 2 h at RT. After washing the plates, detection antibody was added to all wells and incubated for 1 h. Plates were then washed and filled with 100 µL Reading Buffer (MSD) before analysis on the MSD Instrument (Quickplex SQ120, MSD). Protein concentration were measured as pg/mL. Data analysis was performed using MSD Discovery Workbench 4.0 software (Meso Scale Diagnostics).

#### Study protocol—extracellular vesicle characterization

Both the modified protocol which was used as well as extracellular vesicle characterization are described in detail in two previously published papers (specifically in the Supplemental Materials of Zhang et al.)^32,33^. Easy access to this data in a nice structured way can also be obtained via the EV-track that was created with EV-ID: EV200044.

#### Study protocol—coronary artery calcium scoring

A previously developed algorithm was used to determine CAC scores on the low-dose, non-ECG-triggered, attenuation correction CT (LDACT) images acquired during MPI^34^. Scans were acquired with 120 kVp on a hybrid scanner (Biograph CT Flow 64-Slice scanner, Siemens Healthcare, Knowxville, Tennessee). Detailed information on both the scanning protocol for MPI as well as CAC score measurements has been published before^30,35^. In short, the developed algorithm first detects and excludes the lungs to identify the region of interest, on the LDACT. In the identified volume, the algorithm analyzes voxels above the standard intensity level threshold of 130 Hounsfield Units using two subsequent convolutional neural networks. The first network identifies candidate CAC voxels and assigns them a label of the coronary artery they reside in, while the second network identifies true CAC among the candidate CAC voxels. Finally, the identified CAC voxels are quantified using the per artery and total Agatston scores. As this method is not (yet) able to distinguish between CAC and a coronary stent, all patients with a history of coronary revascularization were excluded.

### Statistical analysis

Continuous variables are summarized as mean ± standard deviation (SD) or median with interquartile range [IQR] depending on the distribution. Categorical variables are shown as number with corresponding frequencies. The distribution of all potential confounding variables^36^, CAC scores, and biomarkers were assessed and transformation was performed achieve normal distributions. Levels of OPG were standardized after logarithmic transformation. Patients with levels of OPG > 3 SD were considered as influential outliers and removed from the dataset.

For informative purpose and to correct for all possible confounders, associations between a wide range of cardiovascular risk factors and levels of OPG in plasma and both EV subpopulations were assessed. The continuous association between levels of OPG and the (logarithmically transformed) CAC score were assessed with Spearman’s correlation coefficient. Spearman’s correlation was used instead of Pearson since not all assumptions for Pearson’s correlation were met since no linearity and homoscedasticity was found between the variables. To assess this association in more detail we performed an ordered regression analysis between OPG levels and categories of CAC scores. For this CAC scores were divided in five commonly used categories: 0–9; 10–99; 100–399; 400–999 and > 1000^26,37^. Next to the univariable associations, adjusted ordered regression analysis were performed with two sets of confounders: (1) Age + sex and (2) a parsimonious set of variables. For this parsimonious set of variables we selected general cardiovascular risk factors and variables that were significantly associated with OPG in the regression analysis. The full set of variables contained: age, male sex, smoking, diabetes mellitus, hypercholesterolemia, a family history of CAD, known history of CAD, use of aspirin, statins or betablockade. After model reduction, using a stepwise backward method based on AIC this resulted in a final parsimonious model containing: age, male sex, hypertension, smoking, diabetes mellitus and a history of CAD. In addition, to assess if OPG in plasma and both EV subpopulation showed complementary information they were added all together in a final model.

To provide insight in the potential clinical use we investigated the discriminative ability of levels of OPG in plasma, EV-TEX and EV-LDL to detect significant CAC defined as a CAC score > 10 with logistic regression analysis^20^. After internal validation with bootstrapping techniques C-statistics with corresponding confidence intervals were obtained. All analyses were performed with R Studio Version 1.1.456 (R Foundation for Statistical Computing, Vienna, Austria).

## Results

In total 1265 patients were included in the MYOMARKER cohort study. Seventeen patients were incorrectly included in the study and therefore removed, another 500 patients were excluded because of a history of coronary revascularization, and in six patients there was not enough blood left to perform the analysis. This led to a study population of 742 patients (mean age 67 years, 50.5% male) who are the subject of this manuscript (Table 1). Many patients were overweight with a mean BMI of 27.6, 62% were known with hypertension and half of the patients suffered from hypercholesterolemia, and previous CVD was seen in 75.6%. With regards to medication use, aspirin, statins, betablockade and ACE- or angiotensin-II-inhibition was seen in nearly 50% of the patients. Supplemental Table 1 shows the distribution of cardiovascular risk factors between sexes, despite age (women tend to be slightly older in this study) no statistical differences were seen.

**Table 1:** Baseline characteristics.

	Overall
n	742
**Demographics**	
Age (years)	67 ± 10
Male sex (%)	375 (50.5)
BMI	27.6 (5.2)
**Risk factors**	
Current smoking	145 (19.5)
Diabetes mellitus	139 (18.7)
Hypertension	460 (62)
Hypercholesterolemia	376 (50.7)
Familial coronary artery disease	179 (24.1)
**Medical history**	
Cardiovascular disease	561 (75.6)
Coronary artery disease	53 (7.1)
Heart failure	37 (5.0)
Atrial fibrillation	119 (16.0)
Ischemic CVA	31 (4.2)
**Drug therapy**	
Aspirin	302 (40.7)
P2Y12-inhibitors	53 (7.1)
Anti-coagulants	140 (18.9)
Statin	349 (47.0)
ACE/AT-inhibitor	326 (43.9)
Betablockade	336 (45.3)

Values are shown as mean ± SD or frequency with corresponding percentages.

*CVA* cerebrovascular accident, *AT* Angiotensin II.

### Association between OPG and cardiovascular risk factors

Bivariate correlations between levels of OPG in plasma and both EV-subpopulations and cardiovascular risk factors are provided in Table 2. In all three groups higher age, diabetes, hypertension and betablockade use were associated with higher levels of OPG, while male sex, and a family history of CAD were associated with lower levels of OPG. Hypertension was associated with higher OPG levels in plasma and the EV-LDL subpopulation.

**Table 2:** Bivariate correlations between OPG levels and cardiovascular risk predictors.

	EV-LDL OPG		EV-TEX OPG		Plasma OPG	
	Beta	p value	Beta	p value	Beta	p value
**Variable**						
Age	0.05	**< 0.001**	0.03	**< 0.001**	0.05	**< 0.001**
Male sex	− 0.32	**< 0.001**	− 0.26	**< 0.001**	− 0.33	**< 0.001**
Smoking	− 0.07	0.42	0.03	0.70	0.01	0.88
Diabetes	0.26	**< 0.01**	0.32	**< 0.001**	0.44	**< 0.001**
Hypertension	0.22	**< 0.01**	0.10	0.14	0.22	**< 0.01**
Hypercholesterolemia	0.04	0.56	0.07	0.27	0.12	0.09
Familial CAD	− 0.29	**< 0.001**	− 0.21	**< 0.01**	− 0.24	**< 0.01**
History of CAD	0.09	0.51	− 0.08	0.54	0.07	0.59
Ascal use	0.04	0.59	0.04	0.60	0.03	0.69
Statin use	0.14	**0.04**	0.01	0.89	0.10	0.16
Betablockade use	0.28	**< 0.001**	0.19	**< 0.01**	0.32	**< 0.001**

All biomarkers were transformed depending on their original distribution and standardized.

*EV* extracellular vesicle, *EV-LDL and EV-TEX* both different subpopulation of EVs, *CAD* coronary artery disease.

Bold indicates p value < 0.05.

### Association between OPG and CAC

Valid CAC scores were derived in 720 patients. There were 184 patients with no significant CAC < 10 (25.6%), 124 (17.2%) had mild CAC (10–99), 159 (22.1%) moderate (101–399), 110 (15.2%) severe (> 400) and 143 (19.9%) had extensive CAC (> 1000). Supplemental Table 1 shows CAC scores were significantly higher in men compared to women. Across the OPG-plasma, LDL and TEX measurements in total 15 measurements failed and were therefore reported as missing values. None of the values were considered as influential outliers. In Supplemental Table 2 the untransformed levels of OPG are summarized for each predefined category of CAC. In general levels of OPG gradually increased with increasing CAC scores. Supplemental Fig. 1 provides boxplots comparing levels of OPG for EV-LDL, EV-TEX and plasma between the different categories. No significant differences were found between moderate, severe and extensive CAC.

**Figure 2:**
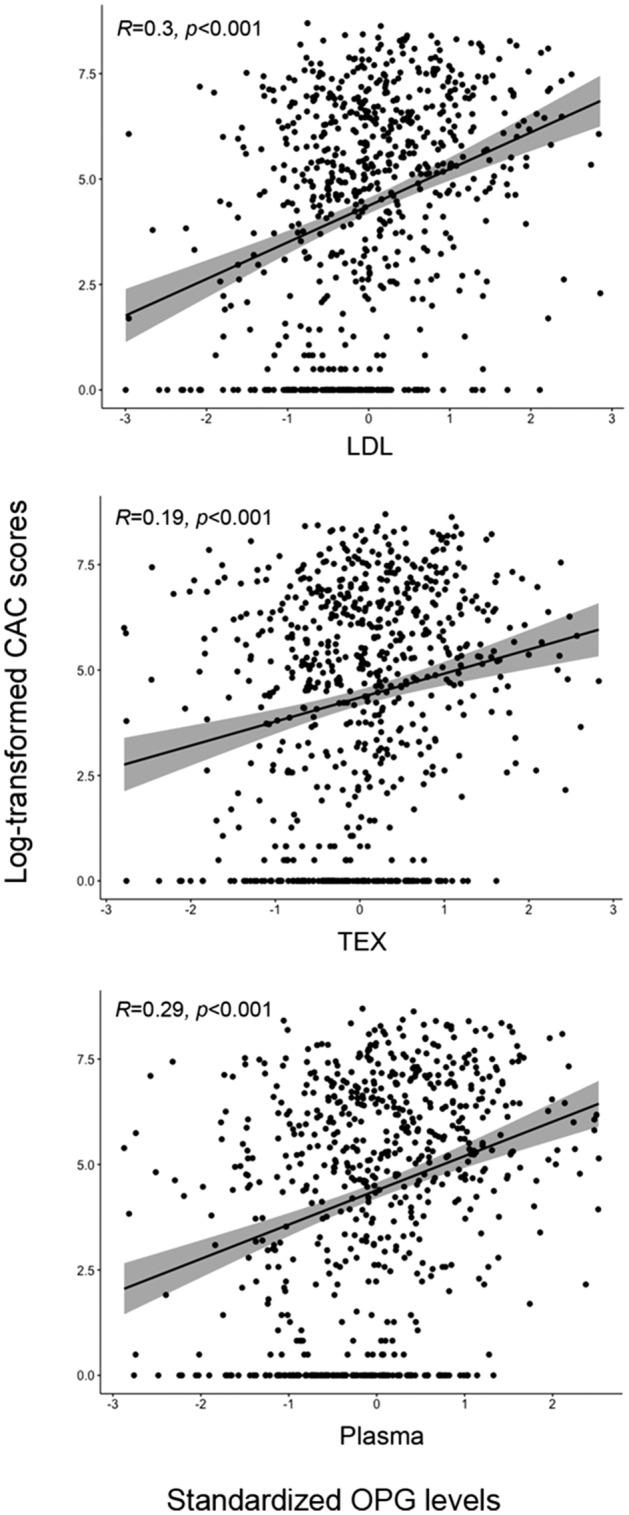
Correlations between standardized levels of OPG and CAC scores in EV-LDL, EV-TEX and plasma CAC scores were logarithmically transformed. *p* values correspond to spearman’s coefficient.

We assessed the associations between levels of OPG in plasma, EV-LDL and EV-TEX and CAC scores. Figure 2 shows that this association was significant for plasma and both EV-subpopulations, all with *p* values < 0.001. The strongest associations were found for EV-LDL with R: 0.3, and plasma, R: 0.29, compared to the weaker association for EV-TEX, R: 0.19.

More in depth analysis was performed with an ordered regression analysis to find associations between levels of OPG and categorical CAC scores, the results of this analysis can be found in Table 3. EV-LDL (odds ratio (OR) 1.81, 95% confidence interval (CI) 1.56–2.10), EV-TEX (OR 1.40; 95% CI 1.21–1.62) and plasma levels of OPG (OR 1.66; 95% CI 1.44–1.91) were significantly associated with categorical CAC scores. The associations remained significant after adjustment for only age and sex as well as full adjustment including cardiovascular risk factors (EV-LDL OR 1.51; 95% CI 1.28–1.80, EV-TEX OR 1.19; 95% CI 1.02–1.40 and plasma OPG OR 1.38; 95% CI 1.16–1.63).

**Table 3:** Ordered regression analysis of OPG levels and categorical CAC scores.

	Univariable	*p* value	Age + sex adjusted	*p* value	Full adjusted	*p* value
EV-LDL OPG	1.85 (1.60–2.15)	< 0.001	1.60 (1.35–1.91)	< 0.001	1.51 (1.27–1.80)	< 0.001
EV-TEX OPG	1.42 (1.23–1.65)	< 0.001	1.27 (1.08–1.49)	< 0.001	1.21 (1.02–1.42)	0.024
Plasma OPG	1.69 (1.47–1.96)	< 0.001	1.49 (1.27–1.77)	< 0.001	1.39 (1.17–1.65)	< 0.001

Full adjusted model included: age, sex, smoking, hypertension, diabetes mellitus and a previous history of coronary artery disease. Outcome variable was categorized in five classes: 0; 1–99; 100–399; 400–999 and > 1000.

*EV* extracellular vesicle, *EV-LDL and EV-TEX* different subpopulations of EVs.

### Discriminatory ability of OPG to detect significant CAC

To assess if OPG plasma levels and EV-derived OPG levels provide additional information OPG levels in plasma and EVs were added together in the parsimonious model. In this model only EV-derived OPG in the LDL-subpopulation remained significantly associated with CAC (OR 1.18; 95% CI 1.05–1.17), while EV-derived OPG in the TEX subpopulation (OR 0.98; 95% CI 0.79–1.21) and OPG plasma levels (OR1.18; 95% CI 0.93–1.52) were no longer significant predictors of CAC. To gain more insight in the potential discriminatory ability of OPG C-statistics were obtained (Table 4). Modest discrimination to predict presence of CAC (defined as CAC > 10) was seen for both plasma OPG and EV-LDL (C-statistics both 0.67), C-statistic for EV-TEX was lower with 0.63.

**Table 4:** C-statistics for OPG to detect significant CAC.

	CAC > 10
	C statistic (95% CI)
EV-LDL OPG	0.67 (0.63–0.71)
EV-TEX OPG	0.63 (0.59–0.65)
Plasma OPG	0.67 (0.65–0.70)

CAC > 10 refers to the clinical scenario with a binary outcome for the coronary artery calcium score defined as < 10 or > 10.

*EV* extracellular vesicle, *EV-LDL and EV-TEX* different subpopulations of EVs.

## Discussion

In this study the association between levels of OPG in EVs and plasma were studied in a large symptomatic population suspected of CCS. We report three major findings, (1) we showed plasma levels of OPG as an independent predictor of CAC scores in a symptomatic population (OR 1.39; 95% CI 1.17–1.65). (2) We reported the first data on EV-derived OPG and the relation with CAC. Additional to plasma OPG, high levels of both EV-LDL OPG (OR 1.51; 95% CI 1.27–1.80) as well as EV-TEX OPG (OR 1.21; 95% CI 1.02–1.42) were independently associated with a higher CAC score category. (3) We assessed the clinical potential of plasma OPG, and EV-derived OPG to correctly stratify patients to their corresponding CAC risk-category. The C-statistics to predict significant CAC (> 10) were only modest.

### OPG and cardiovascular risk factors

We found a positive association between age and OPG, which was also observed in a substudy of the CLARICOR trial^38^. Since atherosclerosis itself is highly age-dependent, the association between OPG and age might be explained by this. Higher levels of OPG are consistently found in women compared to men, the CLARICOR trial showed that women had, irrespective of diabetes or statin use 15% higher levels of OPG. A nested case–control study in the EPIC Norfolk trial showed higher levels of OPG in women compared to men for both cases (coronary event or death from coronary cause) and controls (healthy individuals)^39^. Both studies suggest biological difference in the levels of OPG between sexes but not directly in their association with disease, in our study we found the same result. Other associations with sex, diabetes, hypertension, family history with CAD and the use of beta blockade, were all consistent with existing literature^38,40^. Interestingly, we did not find an association between statin use and OPG, whereas other studies did^38,41,42^. The studies of Kadoglou et al.^41^ and Davenport et al.^42^ were both small studies involving different study populations compared to our study which might explain the difference. However, the CLARICOR trial was also a large study > 3000 participants and the percentage of patients on statin therapy was comparable to our study, approximately 40%. We have no clear explanation for this difference.

### OPG and VC

We found OPG to be an independent predictor of CAC scores. This result is in agreement with previous studies showing the same association in the general population^28,29^ as well as asymptomatic patients with established CVD^26,43–45^. We now show that high levels of OPG are also associated with CAC in a symptomatic chest pain cohort. Additionally, we investigated the association of circulating EV-derived OPG with CAC for the first time. High levels of EV-derived OPG were also associated with high CAC scores, just as plasma levels of OPG. The positive association for both plasma OPG and circulating EV-derived OPG with CAC suggests that they probably both just mirror the same underlying processes^13,18,28^. Two decades ago Jono et al. was the first to report that levels OPG were associated with the degree of CAD measured with coronary angiography^46^. OPG was also found as predictor for progression of carotid atherosclerosis^13^. Several animal studies also showed that in the absence of OPG more VC of the vessel wall is seen^8–10^. Interestingly, Morony et al. showed that administration of OPG (and thus high levels of OPG) in mice deficient for the LDL receptor, led to less calcification of plaques^10^. However, they did not find a decrease in number of plaques, nor lower cholesterol levels in these mice. This might indicate OPG could have a specific role in calcification of the plaques rather than preventing the atherosclerotic process itself. This finding is in contrast to the consistent finding of high levels of OPG in human and its association with MACE. Other groups have also suggested that high levels of OPG are a simple epiphenomenon of inflammation as result of atherosclerosis^47–49^.

Previous studies showed VSMC derived EVs to be present in calcified plaques and they also showed OPG in these EVs^50^. In normal physiological conditions VSMCs are contractile and secrete EVs that regulate the micro-environment which enable phenotypical changes^17^. The secreted VSMC-derived EVs contain calcification inhibitors, such as OPG and matrix GLA-protein^18^. As a result of cellular stress and mineral imbalance VSMCs become less contractile and start to form calcified EVs^51^. Calcified EVs tend to aggregate, form microcalcification and increase calcification in existing plaques^52^. In vulnerable plaques this could lead to rupture, whereas it could also further stabilize a calcified plaque under a thick fibrous cap^52^. We did not find a different association between EV-derived OPG levels and CAC scores compared to plasma OPG. This suggests that EV-derived levels of OPG have a similar function as plasma OPG. The correlation between the EV-derived OPG levels and plasma OPG were statistically significant (LDL R: 0.75 P < 0.001 and TEX R: 0.66, P < 0.001) emphasizing this hypothesis (data not shown). Circulating EV-derived OPG represents a more general view of atherosclerosis itself rather than being involved in the actual process of VC like VSMC-derived EVs are.

### Discriminatory ability of OPG

We showed a moderate correlation between levels of OPG and CAC, additionally, we assessed whether a single OPG measurement in plasma or EVs has the clinical potential to predict the presence of CAC scores (defined as CAC > 10). A cut-off value of 10 was used, since the aim of this analysis was to see if OPG levels have potential to distinguish between patients at risk for future event (presence of CAC) and those without (CAC < 10). If so, this could have immediate clinical implications. After internal validation with bootstrapping techniques, we found a C-statistic to detect CAC > 10 for plasma OPG of 0.67, values for EV-derived OPG were comparable (EV-LDL: 0.67 and EV-TEX: 0.63). One other study investigated the discriminatory ability of a single OPG measurement to predict high CAC scores (> 400) in healthy participants^53^. With C-statistics to detect CAC > 1, CAC > 10 and CAC > 400 of 0.5 they concluded OPG was not able to identify healthy participants with significant CAC scores. Although our C-statistics to detect CAC > 10 were also modest, the discriminatory ability of OPG was clearly better in symptomatic patients compared to healthy participants. To assess the added value of EV-derived OPG, we analyzed EV-OPG and plasma OPG together in the final parsimonious model, no added value was observed. It is therefore likely that EV-derived OPG represents the same process as plasma derived OPG. The modest correlation as well as the cross-sectional, nature of this study makes it quite hard to determine the potential clinical importance of our findings. It remains to be elucidated what the exact role of OPG in the pathophysiology of vascular calcification is. For this, the etiologic relation should be studied in more detail and eventually a clinical impact study would be the next step.

### Limitations

Several limitations merit consideration. Because of the retrospective character of the study, we were not able to investigate whether levels of OPG would have changed clinical decision making by the treating physician. The study remains limited to only one center, which might influence the generalizability of the results. In this study patients with previous coronary revascularization were excluded but the clinical relevance of CAC determination in these patients is limited anyway. Compared to general population studies the sample we studied is only modest (N = 742).

## Conclusion

Increased levels of OPG in plasma were independently associated with CAC scores. High plasma OPG and EV-OPG levels were associated with high CAC scores. Our findings suggests that plasma and EV-derived OPG seem to mirror the same underlying pathophysiological process.

## Supplementary Information

Supplementary Information.

## Data Availability

The datasets generated during and/or analysed during the current study are available from the corresponding author on reasonable request.
