# Supplementary material for: High levels of osteoprotegerin are associated with coronary artery calcification in patients suspected of a chronic coronary syndrome
Source: Sci Rep. 2021 Sep 23;11:18946. doi: 10.1038/s41598-021-98177-4 (PMC8460823; doi:10.1038/s41598-021-98177-4)
Supplement: Supplementary file 1 — Supplementary Information. [file 41598_2021_98177_MOESM1_ESM.docx]

**Supplemental materials**

| **Supplemental table 1. Cardiovascular risk factors stratified on sex** | | | |
| --- | --- | --- | --- |
|  | **Men = 375** | **Women = 367** | *P-value* |
| Age, years | 66.21 (10.24) | 68.57 (10.05) | 0.002 |
| BMI | 27.79 (4.76) | 27.38 (5.68) | 0.276 |
| Known coronary artery disease | 34 (9.1) | 19 (5.2) | 0.056 |
| **Risk factors** |  |  |  |
| Current smoking | 70 (18.7) | 75 (20.4) | 0.606 |
| Diabetes Mellitus | 76 (20.3) | 63 (17.2) | 0.323 |
| Hypertension | 235 (62.7) | 225 (61.3) | 0.760 |
| Hypercholesterolemia | 183 (48.8) | 193 (52.6) | 0.338 |
| Familial coronary artery disease | 82 (21.9) | 97 (26.4) | 0.172 |
| **Coronary calcium** |  |  |  |
| Coronary artery calcium score | 825.91 (1139.03) | 308.72 (528.41) | <0.001 |
| Log transformed CAC score | 4.98 (2.65) | 3.75 (2.58) | <0.001 |
| CAC = Coronary artery calcium. | | | |

| **Supplemental table 2. Levels of osteoprotegerin stratified on CAC categories** | | | | | |
| --- | --- | --- | --- | --- | --- |
|  | **CAC 0-9** | **CAC 10-99** | **CAC 100-399** | **CAC 400-999** | **CAC >1000** |
| n | 184 | 124 | 159 | 110 | 143 |
| EV-LDL OPG | 69 [57-82] | 78 [63-92] | 82 [68-111] | 83 [69-103] | 91 [73-119] |
| EV-TEX OPG | 135 [107- 170] | 155 [114-187] | 163 [122-215] | 153 [127-202] | 172 [137-218] |
| Plasma OPG | 666 [560-809] | 753 [614-900] | 824 [659-1052] | 792 [674-1050] | 888 [737-1047] |
| Levels of osteoprotegerin are shown as median [Interquartile range]. Assay unit is pg/ml. EV=Extracellular Vesicle, EV-LDL and TEX respresent different subpopulations. CAC=Coronary artery calcium | | | | | |


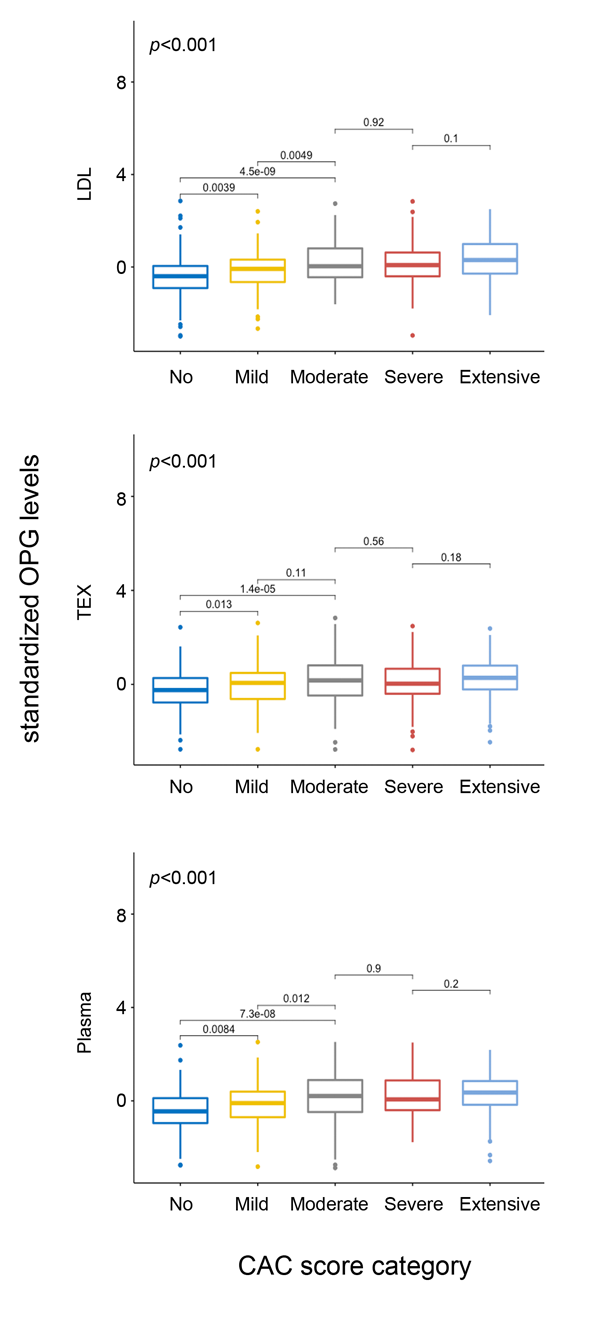


Supplemental figure 1. Standardized levels of OPG per category of CAC score. No = CAC score 0-9, Mild = CAC score 10-99; Moderate = CAC score 100-399, Severe = CAC score 400-999; Extensive = CAC score >1000. *p* values <0.05 were considered significant.
